# Impact of interstitial lung disease on the survival of systemic sclerosis with pulmonary arterial hypertension

**DOI:** 10.1038/s41598-022-09353-z

**Published:** 2022-03-28

**Authors:** Alfredo Guillén-Del-Castillo, Manuel López Meseguer, Vicent Fonollosa-Pla, Berta Sáez Giménez, Dolores Colunga-Argüelles, Eva Revilla-López, Manuel Rubio-Rivas, Maria Jose Cristo Ropero, Ana Argibay, Joan Albert Barberá, Xavier Pla Salas, Amaya Martínez Meñaca, Ana Belén Madroñero Vuelta, Antonio Lara Padrón, Luis Sáez Comet, Juan Antonio Domingo Morera, Cristina González-Echávarri, Teresa Mombiela, Norberto Ortego-Centeno, Manuela Marín González, Carles Tolosa-Vilella, Isabel Blanco, Pilar Escribano Subías, Carmen Pilar Simeón-Aznar, RESCLE Consortium, Águeda Aurtenetxe Pérez, Joan Albert Barberá, Elvira Barrios Garrido-Lestache, Pedro Bedate Díaz, Isabel Blanco, José Manuel Cifrián, Maria Jose Cristo Ropero, Juan Antonio Domingo Morera, Laura Dos Subirá, Teresa Elías Hernández, Pilar Escribano Subías, Francisco José García Hernández, Juan Gil Carbonell, Ariadna González Segovia, Tamara Hermida Valverde, Idaira Fámara Hernández Baldomero, Ignacio Hernández-González, Julia Herrero Huertas, Luis Jara Palomares, Josefa Jiménez Arjona, Antonio Lara Padrón, María Lázaro-Salvador, Manuel López Meseguer, Marta López-Ramón, Raquel López-Reyes, Manuela Marín González, Amaya Martínez Meñaca, Francisco Javier Mazo Etxaniz, Teresa Mombiela, Virginia Naranjo Velasco, Remedios Otero Candelera, Isabel Otero González, Eva Revilla-López, Beatriz Rodríguez Lozano, María Jesús Rodríguez Nieto, Joaquín Rueda Soriano, Berta Sáez Giménez, Belén Safont, Ernest Sala Llinas, Laura Sebastián, Javier Segovia Cubero, María Teresa Subirana Domenech, REHAP Consortium, Ana Argibay, Maria Baldà Masmiquel, Eduardo Callejas Moraga, Antonio-J. Chamorro, Dolores Colunga-Argüelles, Vicent Fonollosa-Pla, Mayka Freire, Cristina González-Echávarri, Alfredo Guillén-del-Castillo, Maria Teresa Herranz Marín, Ana Belén Madroñero Vuelta, Adela Marín Ballvé, Norberto Ortego-Centeno, Melany Pestaña Fernández, Xavier Pla Salas, Ignasi Rodríguez Pintó, Manuel Rubio-Rivas, Luis Sáez Comet, Gonzalo Salvador Cervelló, Carmen Pilar Simeón-Aznar, José Antonio Todolí Parra, Carles Tolosa-Vilella, Luis Trapiella, José Antonio Vargas Hitos

**Affiliations:** 1 Unit of Autoimmune Diseases, Department of Internal Medicine, Hospital Universitario Vall d’Hebron, Barcelona, Spain; 2 Pneumology Department, Hospital Universitario Vall d’Hebrón, Passeig Vall d’Hebron 119-129, 08035 Barcelona, Spain; 3 Physiology Department, Universitat Autònoma de Barcelona, Barcelona, Spain; 4 Department of Internal Medicine, Hospital Universitario Central de Asturias, Oviedo, Asturias Spain; 5 Unit of Autoimmune Diseases, Department of Internal Medicine, Hospital Universitario de Bellvitge-IDIBELL, L’Hospitalet de Llobregat, Barcelona, Spain; 6 Pulmonary Hypertension Unit, Cardiology Department of Hospital, Universitario, 12 de Octubre, Madrid, Spain; 7 Unit of Systemic Autoimmune Diseases and Thrombosis, Department of Internal Medicine, Complejo Hospitalario Universitario de Vigo, Vigo, Pontevedra Spain; 8 Pulmonary Medicine Department, Hospital Clínic de Barcelona/Institut d’Investigacions Biomèdiques August Pi i Sunyer (IDIBAPS), Barcelona, Spain; 9 Centro de Investigación Biomédica en Red de Enfermedades Respiratorias (CIBERES), Madrid, Spain; 10 Unit of Systemic Autoimmune Diseases, Department of Internal Medicine, Consorci Hospitalari de Vic, Vic, Barcelona Spain; 11 Pneumology Department, Hospital Universitario Marqués de Valdecilla, Santander, Cantabria Spain; 12 Department of Internal Medicine, Hospital General San Jorge, Huesca, Spain; 13 Cardiology Department, Hospital Universitario de Canarias, Santa Cruz de Tenerife, Spain; 14 Department of Internal Medicine, Hospital Universitario Miguel Servet, Zaragoza, Spain; 15 Pneumology Department, Hospital Universitario Miguel Servet, Zaragoza, Spain; 16 Autoimmune Diseases Research Unit, Department of Internal Medicine, Biocruces Bizkaia Health Research Institute, Hospital Universitario Cruces, University of the Basque Country, Barakaldo, Spain; 17 Cardiology Department, Hospital Universitario Gregorio Marañón, Madrid, Spain; 18 Inst Invest Biosanitaria Ibs Granada, Department of Internal Medicine, Unit of Systemic Autoimmune Diseases, Hospital Universitario San Cecilio, Granada, Spain; 19 Department of Medicine, Facultad de Medicina, Hospital Universitario San Cecilio, Granada, Spain; 20 Pneumology Department, Hospital Clínico Universitario de Valencia, Valencia, Spain; 21 Department of Internal Medicine, Parc Taulí, Hospital Universitario, Sabadell, Barcelona Spain; 22 Centro de Investigación Biomédica en Red de Enfermedades Cardiovasculares (CIBERCV)/Instituto de Salud Carlos III, Madrid, Spain; 23 Pneumology Department, Hospital Universitario Basurto, Bilbao, Spain; 24 Cardiology Department, Hospital Universitario Rey Juan Carlos, Móstoles, Madrid, Spain; 25 Pneumology Department, Hospital Universitario Central de Asturias, Oviedo, Spain; 26 Unitat Integrada de Cardiopaties Congènites de l’Adolescent i de l’Adult Vall d’Hebron-Sant Pau, Department of Cardiology, Vall d’Hebron University Hospital and CIBERCV, Barcelona, Spain; 27 Unidad Médico-Quirúrgica de Enfermedades Respiratorias, Instituto de Biomedicina de Sevilla (IBiS), Pneumology Department, Hospital Universitario Virgen del Rocío, Sevilla, Spain; 28 Internal Medicine Department, Hospital Universitario Virgen del Rocío, Sevilla, Spain; 29 Pneumology Department, Hospital General Universitario de Alicante, Alicante, Spain; 30 Department of Cardiology, Hospital Universitario Puerta de Hierro-Majadahonda, Madrid, Spain; 31 Department of Cardiology, Hospital Universitario Río Hortega, Valladolid, Spain; 32 Pneumology Department, Hospital Universitario Virgen del Rocío, Sevilla, Spain; 33 Internal Medicine Department, Hospital Jerez de la Frontera, Jerez de la Frontera, Cádiz, Spain; 34 Cardiology Department, Hospital Virgen de la Salud, Toledo, Spain; 35 Cardiology Department, Hospital Universitario Miguel Servet, Zaragoza, Spain; 36 Pneumology Department, Hospital Universitario y Politécnico La Fe, Valencia, Spain; 37 Pneumology Department, Hospital Universitario A Coruña, A Coruña, Spain; 38 Pneumology Department, Hospital Universitario Fundación Jiménez Díaz, Madrid, Spain; 39 Cardiology Department, Hospital Universitario La Fe, Valencia, Spain; 40 Centro de Investigación Biomédica en Red de Enfermedades Cardiovasculares (CIBERCV), Madrid, Spain; 41 Pneumology Department, Hospital Universitario Son Espases, Palma, Islas Baleares Spain; 42 Pulmonary Medicine Department, Hospital Josep Trueta, Gerona, Spain; 43 Department of Cardiology, Hospital Universitario Puerta de Hierro-Majalahonda, Madrid, Spain; 44 Unitat Integrada de Cardiopaties Congènites de l’Adolescent I de l’Adult Vall d’Hebron-Sant Pau, Department of Cardiology, Vall d’Hebron University Hospital, Barcelona, Spain; 45 Department of Internal Medicine, Hospital Clínico Universitario de Salamanca, Universidad de Salamanca-IBSAL, Salamanca, Spain; 46 Unit of Autoimmune Diseases, Department of Internal Medicine, Hospital Clínico Universitario de Santiago, Santiago de Compostela, A Coruña, Spain; 47 Department of Internal Medicine, Hospital General Universitario J.MMorales Meseguer, Murcia, Spain; 48 Unit of Autoimmune Diseases, Department of Internal Medicine, Hospital Clínico Universitario Lozano Blesa, IIS Aragón, Zaragoza, Spain; 49 Department of Internal Medicine, Hospital Universitario Mútua Terrassa, Terrassa, Barcelona Spain; 50 Department of Internal Medicine, Hospital de Manises, Manises, Valencia Spain; 51 Department of Internal Medicine, Hospital Universitario y Politécnico La Fe, Valencia, Spain; 52 Systemic Autoimmune Diseases Unit, Department of Internal Medicine, Hospital Universitario Virgen de las Nieves, Granada, Spain

**Keywords:** Systemic sclerosis, Respiratory tract diseases

## Abstract

To assess severity markers and outcomes of patients with systemic sclerosis (SSc) with or without pulmonary arterial hypertension (PAH-SSc/non-PAH-SSc), and the impact of interstitial lung disease (ILD) on PAH-SSc. Non-PAH-SSc patients from the Spanish SSc registry and PAH-SSc patients from the Spanish PAH registry were included. A total of 364 PAH-SSc and 1589 non-PAH-SSc patients were included. PAH-SSc patients had worse NYHA-functional class (NYHA-FC), worse forced vital capacity (FVC) (81.2 ± 20.6% vs 93.6 ± 20.6%, P < 0.001), worse tricuspid annular plane systolic excursion (TAPSE) (17.4 ± 5.2 mm vs 19.9 ± 6.7 mm, P < 0.001), higher incidence of pericardial effusion (30% vs 5.2%, P < 0.001) and similar prevalence of ILD (41.8% vs. 44.9%). In individuals with PAH-SSc, ILD was associated with worse hemodynamics and pulmonary function tests (PFT). Up-front combination therapy was used in 59.8% and 61.7% of patients with and without ILD, respectively. Five-year transplant-free survival rate was 41.1% in PAH-SSc patients and 93.9% in non-PAH-SSc patients (P < 0.001). Global survival of PAH-SSc patients was not affected by ILD regardless its severity. The multivariate survival analysis in PAH-SSc patients confirmed age at diagnosis, worse NYHA-FC, increased PVR, reduced DLCO, and lower management with up-front combination therapy as major risk factors. In conclusion, in PAH-SSc cohort risk of death was greatly increased by clinical, PFT, and hemodynamic factors, whereas it was decreased by up-front combination therapy. Concomitant ILD worsened hemodynamics and PFT in PAH-SSc but not survival regardless of FVC impairment.

## Introduction

Systemic sclerosis (SSc) is a rare systemic autoimmune disease characterized by fibrosis of the skin and internal organs and vasculopathy^1,2^]. Pulmonary hypertension (PH)-of which pulmonary arterial hypertension (PAH) is the most frequent form in SSc- and interstitial lung disease (ILD) are the two leading contributing causes of early death^3^. When associated with connective tissue diseases (CTD) like SSc, PAH is classified as Group 1.4 of the PH classification^4^. Both PH and ILD may coexist^5–7^, and when ILD is significant PH is classified as Group 3. However, this classification is challenging and difficult to incorporate into clinical practice with SSc patients. Prevalence of PAH in SSc varies across studies between 5 and 19%^8–12^. Prevalence of clinically relevant ILD is higher with a range from 16 to 47% depending on the definition used^13–15^.

Prognosis of SSc-associated PAH is poor with an annual mortality rate of ~ 30% vs. ~ 10% for the idiopathic form of PAH (IPAH) despite similar hemodynamic features^3,16,17^. Response to PAH therapy is also worse^16,18^. The mortality rate attributable to ILD in SSc patients is ~ 33%^3^. Survival rate is significantly shortened when both pulmonary complications coexist^6,7,19^. Nonetheless, studies on mortality associated with PAH and/or ILD in SSc patients are limited by the reduced size of the populations; thus, nationwide registries are useful in these cases.

RESCLE (*Registro de ESCLErodermia*) is the Spanish registry of SSc patients, and has been running since 2006^20^. The prevalence of PAH confirmed by right heart catheterization (RHC) in this registry is ~ 4%^21,22^. REHAP (*Registro Español de Hipertensión Arterial Pulmonar*) is the Spanish registry of patients with PAH, and was created in 2007^23^. The prevalence of SSc-associated PAH was 9.2%^23^. The objective of this study was to assess the clinical characteristics and prognosis of patients with SSc with or without PAH (PAH-SSc/non-PAH-SSc), and the impact of ILD on PAH-SSc by analyzing both nationwide cohorts.

## Results

At the time of study inclusion, there were 1996 patients enrolled in the RESCLE and 3409 in the REHAP registries. Of these, 1589 (79.6%) RESCLE patients did not have a PAH diagnosis (non-PAH-SSc) and 364 (10.7%) REHAP patients had SSc (PAH-SSc) confirmed on RHC. RHC was only performed in 58 non-PAH-SSc patients ruling out this complication, and specifically in 6 out of 38 patients with sPAP > 40 mmHg by echocardiography. These were the populations analyzed. Autoantibody specificities were available in non-PAH-SSc patients, 687/1413 (48.6%) had anti-centromere antibody, 259/1386 (18.7%) had anti-topoisomerase I antibody and 42/353 (11.9%) had anti-RNA polymerase III antibody.

### Impact of PAH on SSc patients

Table 1 summarizes the baseline demographic, clinical, and echocardiography data of patients according to presence of PAH. Compared to non-PAH-SSc patients, PAH-SSc patients were older, had worse New York Heart Association functional class (NYHA FC) and pulmonary function tests (PFTs) (as assessed by % of predicted forced vital capacity (FVC) and diffusing capacity for carbon monoxide (DLCO)); more patients had FVC/DLCO ≥ 1.4 and even ≥ 1.6. Furthermore, mean systolic pulmonary artery pressure (sPAP) was greater in PAH-SSc patients, more patients presented sPAP > 40 mmHg, any grade or moderate-severe degree of tricuspid regurgitation, pericardial effusion, or lower tricuspid annular plane systolic excursion (TAPSE) values. No differences were observed in the prevalence of ILD. Regarding medical treatment, most PAH-SSc patients (62.6%) received up-front combination therapy while 15.9% non-PAH-SSc patients received specific vasodilators for peripheral vasculopathy. These differences did not change when the population was compared according to the presence or absence of ILD (online supplementary table II and III).

**Table 1:** Baseline demographic, clinical, and echocardiography data of PAH-SSc (REHAP) and non-PAH-SSc patients (RESCLE).

	N	PAH-SSc N = 364	N	Non-PAH-SSc N = 1589	P value
Gender, female, n (%)	364	316 (86.8)	1589	1408 (88.6)	0.366
Age at diagnosis, years, mean (SD)	364	62.7 (12.0)	1589	51.3 (15.5)	** < 0.001**
**NYHA FC, n (%)**	364		667		
I–II		107 (29.4)		612 (91.7)	** < 0.001**
III–IV		257 (70.6)		176 (8.2)	** < 0.001**
ILD on HRCT, n (%)	220	92 (41.8)	939	422 (44.9)	0.408
**Pulmonary function test**					
FVC (%) predicted, mean (SD)	329	81.2 (20.6)	1295	93.6 (20.6)	** < 0.001**
< 60%, n (%)		50 (15.2)		83 (6.4)	< 0.001*
60–< 80%, n (%)		105 (31.9)		218 (17)	< 0.001*
≥ 80%, n (%)		174 (52.9)		994 (76.5)	< 0.001*
DLCO (%) predicted, mean (SD)	280	45.3 (17.7)	1011	79.0 (36.6)	** < 0.001**
DLCO ≤ 55%, n (%)		213 (76.1)		156 (15.4)	** < 0.001**
FVC/DLCO, mean (SD)	270	2.1 (1.0)	1005	1.3 (0.4)	** < 0.001**
FVC/DLCO ≥ 1.6, n (%)		183 (67.8)		184 (18.3)	** < 0.001**
FVC/DLCO ≥ 1.4, n (%)		210 (77.8)		350 (34.8)	** < 0.001**
**Electrocardiogram**					
Arrhythmia/Atrial fibrillation, n (%)	318	27 (8.5)	691	46 (6.7)	0.298
**Echocardiography**					
LVEF (%), mean (SD)	243	64.1 (8.5)	1153	63.7 (6.7)	0.526
sPAP, mmHg, mean (SD)	325	70.0 (21.3)	673	27.5 (9.1)	** < 0.001**
sPAP > 40 mmHg, n (%)		314 (96.6)		38 (5.6)	** < 0.001**
Tricuspid regurgitation, yes, n (%)	304	278 (91.4)	1129	520 (46.1)	** < 0.001**
Mild		124 (40.8)		507 (45.0)	0.216
Moderate		116 (38.2)		13 (1.2)	** < 0.001**^£^
Severe		38 (12.5)		0 (0.0)	** < 0.001**^£^
No		26 (8.6)		609 (53.9)	** < 0.001**^£^
TAPSE, mm, mean (SD)	169	17.4 (5.2)	234	19.9 (6.7)	** < 0.001**
Pericardial effusion, n (%)	297	89 (30.0)	1115	58 (5.2)	** < 0.001**
PAH-targeted treatments at diagnosis	364		1589		
No treatment		17 (4.7)		1337 (84.1)	< 0.001*
Monotherapy		119 (32.7)		176 (11.1)	< 0.001*
Up-front combination		228 (62.6)		76 (4.8)	< 0.001*

Significant values are in bold.

*DLCO* diffusing capacity for carbon monoxide, *FVC* forced vital capacity, *HRCT* high-resolution computed tomography, *ILD* interstitial lung disease, *LVEF* left ventricular ejection fraction, *NYHA* *FC* New York Heart Association functional class, *sPAP* systolic pulmonary artery pressure, *SD* standard deviation, *TAPSE* tricuspid annular plane systolic excursion.

*Statistical significant comparison after Bonferroni correction (*p* < 0.017) or ^£^(p < 0.012).

**Figure 1:**
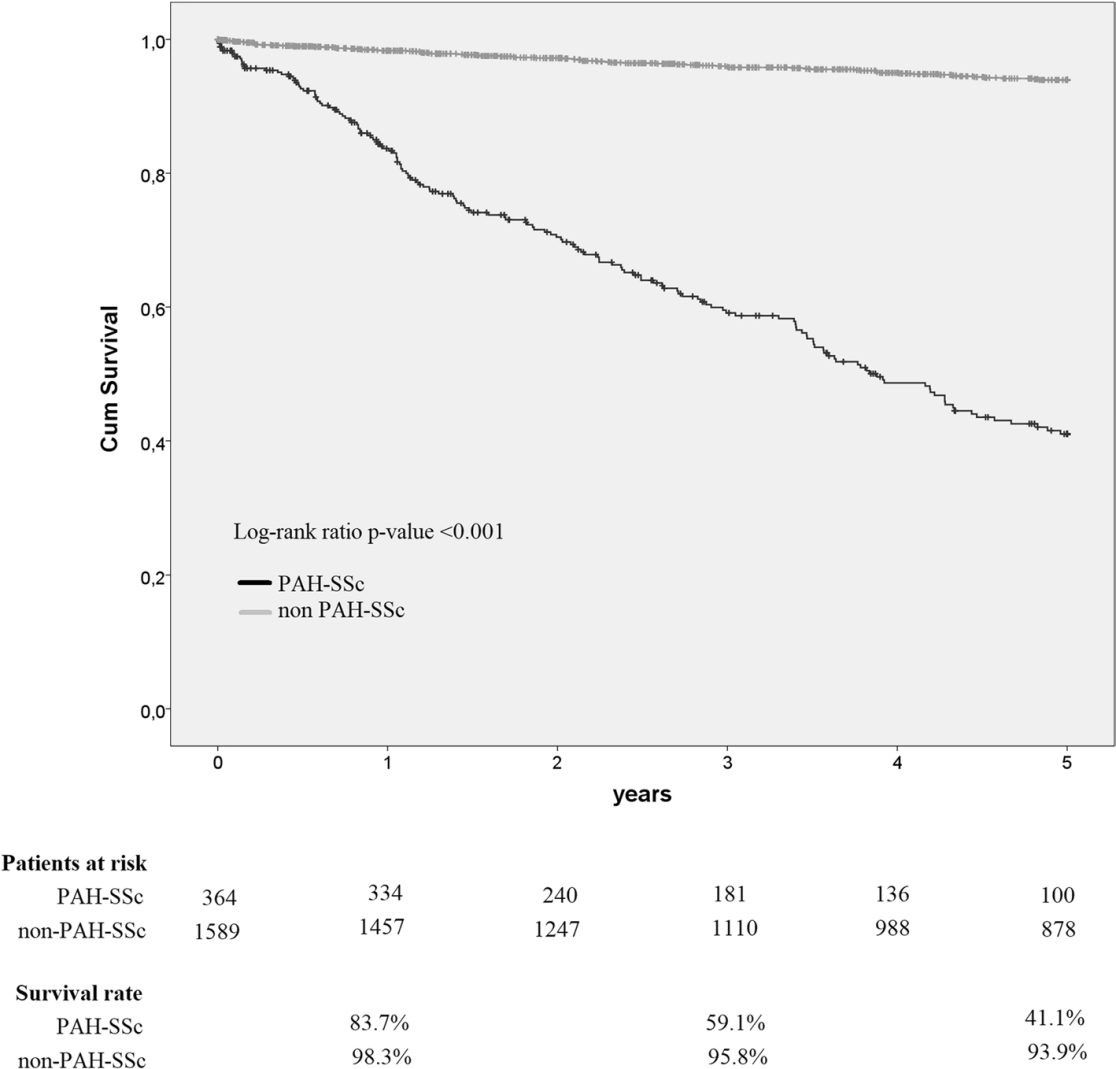
Kaplan–Meier analysis of transplant-free survival in PAH-SSc patients compared with non-PAH-SSc.

Over a median (interquartile range, IQR) follow-up of 2 (1–4) years, 186 (51.1%) PAH-SSc patients died, 14 (3.8%) underwent pulmonary transplantation, and 13 (0.2%) were lost to follow-up. Over a follow-up period of 5 (2–11) years, 185 (11.6%) non-PAH-SSc patients died and 196 (12.3%) were lost to follow-up. The most common causes of death were related to PAH (heart failure and sudden cardiac death) in PAH-SSc patients, while in non-PAH-SSc patients they were related to SSc in 24.3% of cases, malignancies in 17.8%, and others in 29.2% (online supplementary table IV). Kaplan–Meier curves and 1-, 3- and 5-year survival rates for PAH-SSc and non-PAH-SSc patients are shown in Fig. 1. The 5-year survival rate from PAH diagnosis was 41.1% in PAH-SSc patients and 93.9% in non-PAH-SSc patients from SSc diagnosis (P < 0.001).

### Impact of ILD and the severity of FVC impairment on PAH-SSc

**Figure 2:**
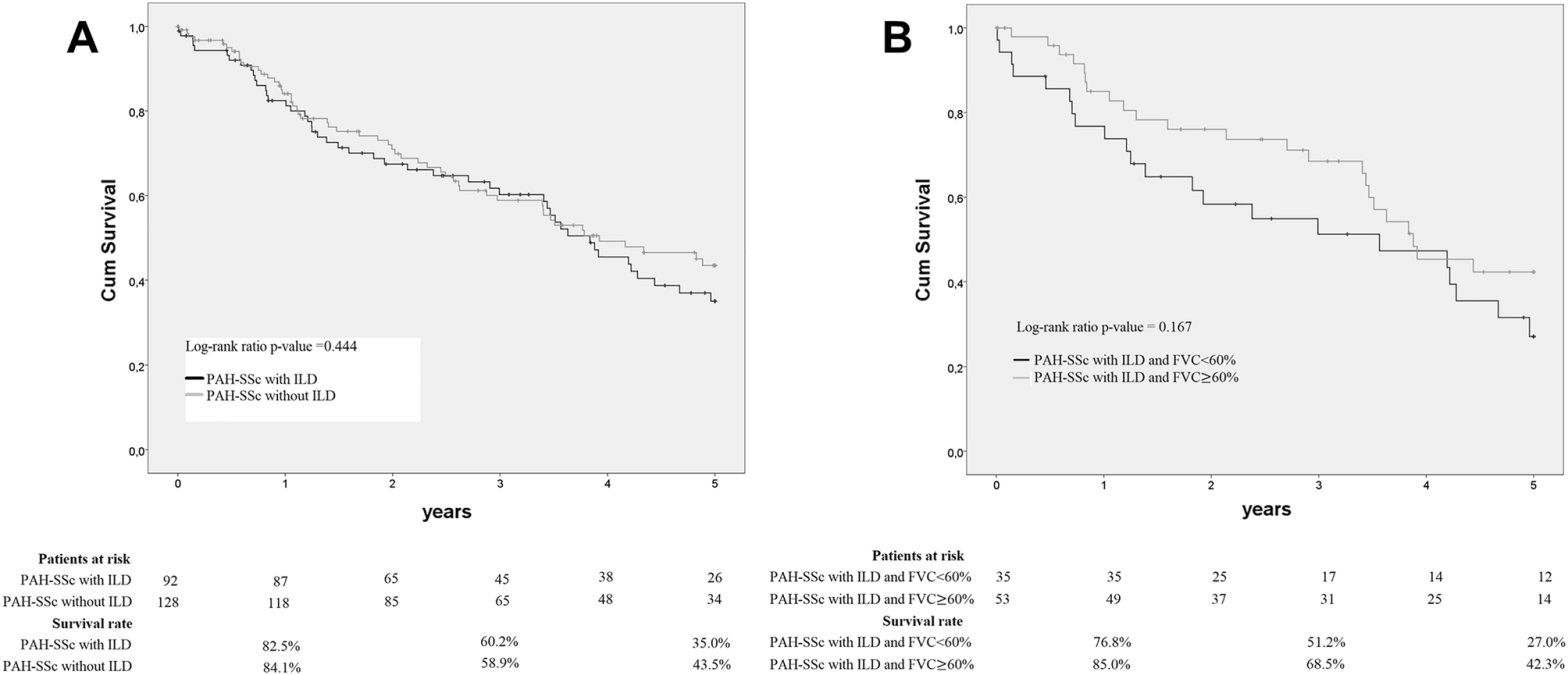
Kaplan–Meier analysis of transplant-free survival in patients with PAH-SSc according to (**A**) presence of ILD and (**B**) severity of the restrictive lung disease.

Of the 220 PAH-SSc patients who had high-resolution computed tomography (HRCT) scans, 92 (41.8%) had ILD. Patients’ characteristics are shown in Table 2. Compared with PAH-SSc without ILD patients, those with concomitant ILD had lower female proportion and more patients presented impaired PFTs, with FVC < 60% and DLCO ≤ 55%. The extent or specific ILD patterns were not available. Nevertheless, in order to estimate the severity according to Goh’s criteria, 48 out of 88 (54.5%) PAH-SSc with ILD patients had extensive disease taking into account FVC < 70%. Right atrial pressure (RAP), cardiac output (CO), cardiac index (CI), and mean pulmonary artery pressure (mPAP) were significantly lower. No significant differences were found on echocardiography, but mean TAPSE was lower. No differences were observed regarding treatment strategies in patients with concomitant ILD and those without. Up-front combination therapy was the most frequent treatment in both cases (59.8% of patients with ILD and 61.7% of patients without ILD). No differences in transplant-free survival were observed in PAH-SSc patients according to the presence of ILD (P = 0.444) (Fig. 2A). Nevertheless, 1-, 3- and 5-year transplant-free survival rates were 82.5%, 60.2%, and 35% vs. 84.1%, 58.9%, and 43.5%, respectively, with a tendency for shorter survival in PAH-SSc patients with concomitant ILD.

**Table 2:** Demographic, clinical, and hemodynamic data of patients with PAH-SSc according to the presence of ILD.

	N	PAH-SSc with ILD N = 92	N	PAH-SSc without ILD N = 128	P value
Gender, female, n (%)	92	75 (81.5)	128	117 (91.4)	**0.040**
Age at PAH diagnosis, years, mean (SD)	92	62.1(11.8)	128	63.8 (11.4)	0.302
**NYHA FC, n (%)**	92		128		
I–II	–	22 (23.9)	–	46 (35.9)	0.076
III–IV	–	70 (76.1)	–	82 (64.6)	0.076
6MWT, meters, mean (SD)	78	284.9 (140.5)	105	281.0 (136.2)	0.851
**Hemodynamics, mean (SD)**					
RAP, mmHg	91	8.0 (4.7)	127	9.7 (4.8)	**0.009**
SvO2, %	53	65.4 (8.1)	72	64.8 (11.4)	0.723
CO, L/min	91	4.0 (1.2)	128	4.4 (1.5)	**0.030**
CI, L/min/m^2^	84	2.4 (0.6)	113	2.6 (0.8)	**0.019**
PVR, Wood units	91	8.2 (5.0)	128	8.9 (4.9)	0.374
mPAP, mmHg	92	38.3 (11.1)	128	43.0 (11.9)	**0.003**
**Pulmonary function test**					
FVC (%) predicted, mean (SD)	88	70.9 (21.9)	113	86.2 (18.6)	** < 0.001**
< 60%, n (%)	–	35 (39.8)	–	6 (5.3)	< 0.001*
60–< 80%, n (%)	–	20 (22.7)	–	39 (34.5)	0.086
≥ 80%, n (%)	–	33 (37.5)	–	68 (60.2)	0.002*
DLCO (%) predicted, mean (SD)	74	39.4 (17.0)	99	49.1 (17.9)	** < 0.001**
DLCO ≤ 55%, n (%)	–	64 (86.5)	–	71 (71.7)	**0.026**
FVC/DLCO, mean (SD)	74	2.2 (1.2)	93	2.0 (0.8)	0.270
FVC/DLCO ≥ 1.6, n (%)	–	48 (64.9)	–	63 (67.7)	0.743
FVC/DLCO ≥ 1.4, n (%)	–	54 (73.0)	–	73 (78.5)	0.467
**Biomarkers, median (IQR)**					
NTproBNP, pg/mL	29	1350 (331–3341)	50	1169 (394–3599)	0.814
BNP, pg/mL	21	255 (80–700)	24	146 (126–422)	0.393
**Electrocardiogram**					
Arrhythmia/atrial fibrillation, n (%)	81	7 (8.6)	119	11 (9.2)	1.000
**Echocardiography**					
LVEF (%), mean (SD)	64	63.9 (8.5)	95	64.3 (8.2)	0.787
sPAP, mmHg, mean (SD)	82	65.5 (20.4)	116	69.6 (22.5)	0.192
sPAP > 40 mmHg, n (%)	–	77 (93.9)	–	114 (98.3)	0.128
Tricuspid regurgitation, yes, n (%)	83	76 (91.6)	106	102 (96.2)	0.217
Mild	–	35 (42.2)	–	40 (37.7)	0.552
Moderate	–	30 (34.9)	–	47 (44.3)	0.297
Severe	–	11 (13.2)	–	15 (14.1)	1.000
No	–	7 (8.4)	–	4 (3.8)	0.217
TAPSE, mm, mean (SD)	44	16.5 (5.3)	60	18.9 (4.8)	**0.018**
Pericardial effusion, n (%)	83	20 (24.1)	104	30 (28.8)	0.509
PAH-targeted treatments at diagno***sis***	92		128		
No treatment	–	4 (4.3)	–	6 (4.7)	1.000
Monotherapy	–	33 (35.9)		43 (33.6)	0.774
Up-front combination	–	55 (59.8)	–	79 (61.7)	0.781

Significant values are in bold.

*BNP* B-type natriuretic peptide, *CI* cardiac index, *CO* Cardiac output, *DLCO* diffusing capacity for carbon monoxide, *FVC* forced vital capacity, *IQR* interquartile range, *ILD* interstitial lung disease, *LVEF* left ventricular ejection fraction, *mPAP* mean pulmonary artery pressure, *NTproBNP* N-terminal pro B-type natriuretic peptide, *NYHA FC* New York Heart Association functional class, *PVR* pulmonary vascular resistance, *RAP* right atrial pressure, *sPAP* systolic pulmonary artery pressure, *SD* standard deviation, *SvO*_*2*_ mixed venous oxygen saturation, *TAPSE* tricuspid annular plane systolic excursion, *6MWT* 6-min walking test.

*Statistical significant comparison after Bonferroni correction (*p* < 0.017).

FVC was available in 88 out of 92 patients with PAH-SSc and ILD. Thirty-five (40%) of these patients had FVC < 60%. Patients’ characteristics are shown in supplementary table V. Patients with FVC < 60% were younger at diagnosis and had lower mean FVC/DLCO compared to their counterparts with FVC ≥ 60%_._ No differences were observed in gender, NYHA FC or 6-min walk test (6MWT), hemodynamics, biomarkers, electrocardiogram, or echocardiographic variables. Up-front combination therapy was the preferred approach in both cases (51.4% in FVC < 60% group and 66.0% in FVC ≥ 60% patients), although there was a trend towards lower use of up-front combination therapy and greater use of monotherapy in patients with FVC < 60% (P = 0.042). For PAH-SSc patients, no differences in survival were found regarding FVC impairment (FVC < 60% vs FVC ≥ 60% patients) (P = 0.167) (Fig. 2B). However, there was a numerical reduction at 1-, 3- and 5-year survival rates in patients with FVC < 60% compared with FVC ≥ 60% (76.8%, 51.2% and 27.0% vs. 85.0%, 68.5% and 42.3%, respectively), with a trend to shorter survival.

### Univariate and multivariate survival analysis in PAH-SSc and non-PAH-SSc patients

For both populations, factors associated to transplant-free survival on univariate analysis are shown in Table 3.

**Table 3:** Factors associated to survival in univariate analyses.

	PAH-SSc		Non-PAH-SSc	
	HR (95% CI)	P value	HR (95% CI)	P value
Gender, female	0.96 (0.63–1.45)	0.846	0.42 (0.29–0.60)	**< 0.001**
Age at diagnosis, years	1.02 (1.00–1.03)	**0.010**	1.08 (1.07–1.09)	**< 0.001**
NYHA FC III–IV^†^	1.98 (1.40–2.80)	** < 0.001**	2.95 (1.78–4.89)	**< 0.001**
ILD on HRCT	1.20 (0.85–1.71)	0.304	1.60 (1.11–2.32)	**0.013**
6MWT, per 10-m increase	0.97 (0.96–0.98)	** < 0.001**	NA	NA
H**emodynamics**				
RAP, per 5-mmHg increase	1.16 (1.01–1.33)	**0.035**	NA	NA
SvO2, per 5%- increase	0.92 (0.84–1.00)	0.052	NA	NA
CO, L/min	0.80 (0.73–0.89)	** < 0.001**	NA	NA
CI, L/min/m^2^	0.68 (0.56–0.82)	** < 0.001**	NA	NA
PVR, Wood units	1.06 (1.04–1.08)	** < 0.001**	NA	NA
mPAP, per 10-mmHg increase	1.16 (1.05–1.28)	**0.004**	NA	NA
**Biomarkers**				
NTproBNP, per 300-pg/mL increas e^‡^	1.02 (1.01–1.03)	** < 0.001**	NA	NA
BNP, per 50-pg/mL increase	1.00 (0.98–1.01)	0.547	NA	NA
FVC, per 10%- predicted increase	0.91 (0.85–0.98)	**0.012**	0.76 (0.70–0.83)	** < 0.001**
DLCO, per 10%- predicted increase	0.82 (0.75–0.91)	** < 0.001**	0.99 (0.98–1.00)	**0.023**
FVC/DLCO ≥ 1.4	1.43 (0.94–2.18)	**0.045**	1.02 (0.63–1.63)	0.949
Arrhythmia/Atrial fibrillation^†^	1.38 (0.83–2.31)	0.219	2.94 (1.78–4.86)	** < 0.001**
**Echocardiography**				
LVEF, per 5% increase	0.94 (0.85–1.04)	0.208	0.82 (0.72–0.93)	**0.002**
sPAP, per 10-mmHg increase ^†^	1.11 (1.05–1.18)	** < 0.001**	1.55 (1.11–2.16)	**0.010**
sPAP > 40 mmHg ^†^	5.18 (1.28–21.01)	**0.021**	2.78 (1.44–5.37)	**0.002**
Tricuspid regurgitation, yes	1.41 (0.78–2.54)	0.251	0.69 (0.49–0.99)	**0.043**
Mild	0.64 (0.47–0.89)	**0.007**	0.69 (0.48–0.99)	**0.041**
Moderate	1.38 (1.01–1.88)	**0.041**	1.08 (0.15–7.75)	0.939
Severe	1.62 (1.07–2.46)	**0.022**	1.44 (1.01–2.06)	**0.043**
TAPSE, ≥ 18 mm*^†‡^	0.60 (0.38–0.94)	**0.026**	–	–
Pericardial effusion	1.40 (1.00–1.96)	0.053	0.42 (0.29–0.60)	** < 0.001**
**PAH-targeted treatments at diagnosis**				
No	1.34 (0.69–2.63)	0.388	0.79 (0.48–1.29)	0.347
Monotherapy	1.30 (0.96–1.75)	0.086	0.77 (0.45–1.31)	0.333
Up-front combination	0.74 (0.56–0.99)	**0.046**	0.95 (0.30–2.97)	0.927

Significant values are in bold.

^†^Parameter not included in the multivariate analysis as it was available in less than 60% of non-PAH-SSc patients.

^‡^Parameter not included in the multivariate analysis as it was available in less than 60% of PAH-SSc patients.

*All death patients in non-PAH-SSc had TAPSE ≥ 18 mm.

The multivariate survival analysis in PAH-SSc patients identified age at diagnosis (hazard ratio (HR) 1.02 [95% CI 1.00–1.03]; P = 0.036), NYHA FC III-IV (HR 1.63 [95% CI 1.10–2.42]; P = 0.015), and pulmonary vascular resistance (PVR) (HR 2.41 [95% CI 1.37–4.25]; P = 0.002) as poor prognostic indicators, whereas DLCO per 10%—predicted increase (HR 0.87 [95% CI 0.78–0.97]; P = 0.009) and up-front combination therapy (HR 0.54 [95% CI 0.38–0.77]; P < 0.001) were the only factors associated to better prognosis (Table 4). The multivariate analysis in non-PAH-SSc patients showed that older age at diagnosis (HR 1.09 [95% CI 1.07–1.11]; P < 0.001) worsened prognosis, while FVC per 10%—predicted increase(HR 0.80 [95% CI 0.72–0.88]; P < 0.001) and DLCO per 10%—predicted increase (HR 0.92 [95% CI 0.85–1.00]; P = 0.048) were associated with greater survival.

**Table 4:** Factors associated to survival in multivariate analyses.

	PAH-SSc			Non-PAH-SSc	
	HR (95% CI)	P value		HR (95% CI)	P value
Age, years	1.02 (1.01–1.03)	**0.033**	Age, years	1.09 (1.07–1.11)	** < 0.001**
NYHA FC III–IV	1.63 (1.10–2.42)	**0.015**	FVC, per 10%-predicted increase	0.80 (0.72–0.88)	** < 0.001**
PVR, wood units	2.41 (1.37–4.25)	**0.002**	DLCO, per 10%-predicted increase	0.92 (0.85–0.99)	**0.048**
DLCO, per 10%-predicted increase	0.87 (0.78–0.97)	**0.009**			
Up-front combination therapy	0.54 (0.38–0.77)	** < 0.001**			

Significant values are in bold.

## Discussion

By linking two nationwide registries this study, the largest conducted to date, further highlights the huge impact that PAH has on SSc patients. Approximately half of the patients were diagnosed with ILD independently of the presence of PAH. In PAH-SSc patients, ILD was found to worsen PFTs and hemodynamics but did not have a direct significant impact on survival, regardless of the severity of the ventilatory restrictive pattern. Older age, worse NYHA FC stages, higher PVR, and reduced DLCO at the time of diagnosis were independently linked to poor prognosis. Current treatment strategies (i.e., greater use of up-front combination therapy) are likely to have had an impact on survival of PAH-SSc patients even when they experienced mild-moderate ILD.

PAH-SSc patients had higher sPAP and tricuspid regurgitation velocity, were older, had worse NYHA FC, lower DLCO and elevated FVC/DLCO ratio (> 1.6 and 1.4), and greater prevalence of pericardial effusion; all of them well known clinical features of SSc-associated PAH. The devastating effect of PAH on SSc was reflected by a reduction of nearly 60% in 5-year transplant-free survival (41.1% vs. 93.9% in non-PAH-SSc patients). Three-year survival rate of PAH-SSc patients in our study (59.1%) was similar to that reported by other registries^9,19,24–28^ and a meta-analysis performed by Lefevre et al.^29^. Only in the large prospective PHAROS study 3-year survival rate was higher (75%)^30^. This has been attributed to earlier diagnosis of PAH as reflected by the greater percentage of patients with NYHA FC I-II (59% vs. ~ 30% in the above-mentioned registries and in our series). Availability of new PAH-targeted therapies and treatment strategy changes during follow-up are also likely to affect survival. However, in the meta-analysis published by Lefevre et al.^29^ survival did not change between studies over time, and disease’s severity at baseline was the most important prognostic factor. Up to 60% of PAH-SSc patients in our series received up-front combination therapy in contrast to the 43% in the French registry and the 34% in the REVEAL registry, both contemporary^24,27^. Subsequent evidence endorsed up-front combination treatment^31^, and current guidelines recommend this strategy for most of the patients^32^. Even though the increased use of up-front combination therapy in our study was not correlated with better global survival compared to the registries mentioned above, this strategy was independently associated with greater survival in our cohort.

Approximately half of the patients with PAH presented ILD, with 40% showing a moderate-severe restrictive ventilatory pattern. PAH-SSc patients with ILD had lower FVC, DLCO, TAPSE, and CI. Recently, Chauvelot et al. analyzed 128 patients from the French prospective PH registry: 66 with SSc-PH-ILD and 62 with SSc-PAH^33^. Patients with SSc-PH-ILD had lower FVC and lower DLCO. Use of first-line PAH-specific therapies was similar in both groups and included endothelin receptor antagonists (80%), phosphodiesterase 5 inhibitors (13%), or a combination of both (6%). Only 3 patients received a prostacyclin analog as initial treatment.

In our study, PAH-SSc patients with ILD and FVC ≥ 60% presented with FVC/DLCO ≥ 1.6 more frequently, indicating a more prominent vascular involvement in this subgroup. The threshold determining the extension of ILD leading to one or another classification PH group is blurred and remains to be defined. Thus, when patients present with precapillary PH and mild ILD they are classified into PH Group 1, and when they have more severe ILD they are classified into PH Group 3 (PH-ILD). Despite this fact, a significant proportion of patients present with intermediate severity of ILD and with different degrees of PH, which make PH classification and the following treatment decision especially challenging.

In this cohort, the worse hemodynamics and pulmonary capacity at PAH-SSc diagnosis associated with concomitant ILD did not turn into worse transplant-free survival, although a trend to higher 5-year survival was observed in patients with lower FVC. Previous studies have reported increased mortality in patients with PAH-SSc and ILD^15,29,34^, but most of them merged PAH-SSc patients with mild ILD (PH group 1) with PH-ILD patients. As in our series, Volkmann et al. reported similar 3-year survival rates in PAH-SSc patients with and without ILD (50% and 60%, respectively)^35^, which were associated with early use of aggressive treatment (i.e., prostanoid therapy was used in 52% of patients with ILD). Recently, Young et al. have described a prospective cohort of 93 patients with ILD, identifying a PH prevalence of 29 (31.2%) with a 3-year survival of 91%^36^. Such optimal survival may be explained by the intensive PH screening program and the extensive use of vasodilator therapy (82.8% of the patients with PH). Conversely, the survival in the French registry was significantly shorter in patients with SSc-PH-ILD compared to those with SSc-PAH. In SSc-PH-ILD patients, the survival rates at 1, 2, and 3 years were 91.9%, 78.8%, and 58.5%, respectively, compared to 95.9%, 91.3%, and 78.6% in SSc-PAH patients (*P* = 0.04)^33^.

A more conservative treatment approach with higher use of PAH monotherapy at diagnosis was observed for patients with ILD and FVC < 60%. This was probably due to safety concerns associated with the use of PAH-targeted therapies in patients with PH-ILD^18^ as these latter patients have traditionally been excluded from PAH clinical trials^37^.

Facing the reality that we still cannot precisely classify PH-SSc when associated with ILD, there is increasing evidence suggesting that early use of pulmonary vasodilator treatment improves outcomes, and nationwide registries confirm a widespread off-label use of these drugs in real life, reflecting that treating PAH is a priority for clinicians irrespective of the severity of ILD. Our results reinforce this idea and indicate that treating PAH-SSc aggressively from onset improves outcomes regardless of the presence of ILD.

In PAH-SSc patients, prognostic factors identified in univariate survival analysis were similar to prior meta-analysis^29^, although lower FVC or DLCO, increased FVC/DLCO ratio ≥ 1.4, and lower use of up-front combination therapies at the time of PAH diagnosis were also identified as indicators of poorer survival. Interestingly, the presence of ILD or reduced FVC were not identified as risk factors in the multivariate analysis, whereas older age, worse NYHA FC, elevated PVR or reduced DLCO, and monotherapy at PAH diagnosis were associated to worse prognosis. Conversely, in the French PH registry only the presence of ILD, chronic kidney disease, and 6-min walk distance at baseline were associated with greater mortality^33^. Concerning the age at PAH diagnosis, the French national study conducted between 2006 and 2017, has described an improvement in survival in patients ≤ 70 years but not in older ones^28^. That may be explained by the higher proportion of patients that, in the later years, has been treated with pulmonary vasodilator up-front combination therapy both in the first 4 months (48.6% vs 25.6%) and throughout the study (64.3% vs 39%). Our results support the use of up-front combination therapy at early PAH diagnosis regardless of age in order to improve transplant-free survival.

Several limitations have to be recognized in the interpretation of this study, some of them (e.g., only using variables common to both registries, not analyzing treatments during follow-up nor the last one reported) have been already noted. RHC was not performed for the selection of non-PAH-SSc patients due to it is an invasive procedure, and it is indicated after cautious doctor’s decision. ILD-targeted therapy was not available in PAH-SSc cohort that may also influence on survival of this patients. Both registries are voluntary, which leads to a lack of information on variables that may impact prognosis. To mitigate this limitation, multivariable analysis was carried out using only variables available in > 60% of patients.

## Conclusion

The largest assessment ever of the impact of PAH on SSc confirms the very relevant clinical and prognostic repercussion of PAH on SSc. When associated with ILD, PAH-SSc presents with worse hemodynamic features and PFTs, but not poorer survival independently of ILD severity. Baseline treatment with pulmonary vasodilator up-front combination therapy was established in a majority of PAH-SSc patients regardless of the presence of ILD and was independently associated with longer survival.

## Materials and methods

### Patients

Study design, inclusion and exclusion criteria, and data collection of RESCLE and REHAP registries have been published elsewhere^20,23^. All methods were carried out in accordance with relevant guidelines and regulations and the study was approved by the Hospital Vall d’Hebron Institutional Review Board [PR(AMI)280/2018]. In brief, RESCLE is a voluntary nationwide registry of patients with SSc diagnosed on the 2013 ACR/EULAR criteria for SSc^38^ and/or on the modified LeRoy and Medsger classification criteria^39^. The onset of scleroderma was defined as the first symptom related to SSc including Raynaud’s phenomenon. Both prevalent and incident non-PAH-SSc patients from RESCLE registry were included in the analysis, and excluding PH-SSc patients. REHAP is also a voluntary nationwide registry designed to prospectively collect exhaustive information on the demographics, management, and outcome of patients newly and previously diagnosed with PAH by RHC^23^. PAH was defined as a mean pulmonary arterial pressure (mPAP) ≥ 25 mmHg at rest with a pulmonary artery wedge pressure ≤ 15 mmHg and pulmonary vascular resistances ≥ 3 Wood units at RHC^32^. For the purposes of this study, only prospectively recruited incident patients with SSc-associated PAH (PAH-SSc) from REHAP registry were included in this analysis.

### Data collected

Baseline data from RESCLE and REHAP registries at the time of diagnosis of SSc and PAH respectively were collected. The following variables, considered potential risk factors for PAH in SSc^40–42^, were common to both registries and included in the analyses: (1) Demographics: age at the time of diagnosis (SSc or PAH) and gender^24,41^; (2) Clinical: New York Heart Association functional class (NYHA FC) and time since SSc diagnosis^20,24,40,41^ (only for RESCLE patients); (3) Pulmonary function test (PFT): predicted forced vital capacity (FVC %), predicted diffusing capacity for carbon monoxide (DLCO %)^24,40,41^ and the FVC%/DLCO% ratio > 1.6^43^. We also evaluated a less restrictive cut-off value of 1.4, which has been associated to PH in patients with ILD^44,45^. ILD was defined as the presence of an interstitial pattern on high-resolution computed tomography (HRCT) in REHAP, and by HRCT or chest x-ray in RESCLE. Comparative analyses were performed only in patients with HRCT-confirmed ILD; (4) Echocardiography assessments: left ventricular ejection fraction (LVEF), systolic PAP (sPAP), degree of tricuspid regurgitation, pericardial effusion, and tricuspid annular plane systolic excursion (TAPSE)^24^; (5) Causes of death, which were homogenized as both registries had different approaches of capturing these data (online supplementary table I). For the definition of independent prognostic factors in PAH-SSc and to analyze the impact of ILD on PAH-SSc, we also selected prognostic variables including 6-min walk test (6MWT), hemodynamic parameters (cardiac output [CO], cardiac index [CI], mean pulmonary artery pressure [mPAP], pulmonary vascular resistance [PVR], right atrial pressure [RAP], and mixed venous oxygen saturation [SvO_2_]), and biomarkers (N-terminal pro B-type natriuretic peptide [NTproBNP] or B-type natriuretic peptide [BNP]).

Patient demographics, clinical variables, cardiac, and pulmonary assessments were prospectively recorded by participating physicians according to a standard protocol. Both registries required all patients to provide written informed consent in order to participate. The Institutional Review Boards of the participating hospitals approved the respective registries.

### Statistical analyses

Continuous variables were summarized as the mean ± SD or the median and interquartile range (IQR) as appropriate and compared using Student’s t-test or Mann–Whitney U test, respectively. Categorical variables were compared using the chi-square and Fisher’s exact tests as appropriate. P values < 0.05 (2-tailed) were considered significant. Bonferroni correction was applied in multiple comparisons. Patients lost to follow-up were censored on the day of their last visit. Time-to-event analyses were performed using the Kaplan–Meier method until date of lung transplantation or death. Transplant-free survival was estimated since the time of SSc diagnosis in non-PAH-SSc patients, and since PAH diagnosis in PAH-SSc patients. Factors associated with worse prognosis were identified using the Cox proportional hazards models. Variables collected in > 60% of patients that were found to be significant in univariate analysis (P < 0.05) were incorporated into a step-wise multivariate model.

## Supplementary Information

Supplementary Information.

## Data Availability

The data that support the findings of this study are available on request from the corresponding author.
