# Author Correction: Impact of interstitial lung disease on the survival of systemic sclerosis with pulmonary arterial hypertension

**DOI:** 10.1038/s41598-022-17525-0

**Published:** 2022-07-28

**Authors:** Alfredo Guillén-Del-Castillo, Manuel López Meseguer, Vicent Fonollosa-Pla, Berta Sáez Giménez, Dolores Colunga-Argüelles, Eva Revilla-López, Manuel Rubio-Rivas, Maria Jose Cristo Ropero, Ana Argibay, Joan Albert Barberá, Xavier Pla Salas, Amaya Martínez Meñaca, Ana Belén Madroñero Vuelta, Antonio Lara Padrón, Luis Sáez Comet, Juan Antonio Domingo Morera, Cristina González-Echávarri, Teresa Mombiela, Norberto Ortego-Centeno, Manuela Marín González, Carles Tolosa-Vilella, Isabel Blanco, Pilar Escribano Subías, Carmen Pilar Simeón-Aznar, RESCLE Consortium, Águeda Aurtenetxe Pérez, Joan Albert Barberá, Elvira Barrios Garrido-Lestache, Pedro Bedate Díaz, Isabel Blanco, José Manuel Cifrián, Maria Jose Cristo Ropero, Juan Antonio Domingo Morera, Laura Dos Subirá, Teresa Elías Hernández, Pilar Escribano Subías, Francisco José García Hernández, Juan Gil Carbonell, Ariadna González Segovia, Tamara Hermida Valverde, Idaira Fámara Hernández Baldomero, Ignacio Hernández-González, Julia Herrero Huertas, Luis Jara Palomares, Josefa Jiménez Arjona, Antonio Lara Padrón, María Lázaro-Salvador, Manuel López Meseguer, Marta López-Ramón, Raquel López-Reyes, Manuela Marín González, Amaya Martínez Meñaca, Francisco Javier Mazo Etxaniz, Teresa Mombiela, Virginia Naranjo Velasco, Remedios Otero Candelera, Isabel Otero González, Eva Revilla-López, Beatriz Rodríguez Lozano, María Jesús Rodríguez Nieto, Joaquín Rueda Soriano, Berta Sáez Giménez, Belén Safont, Ernest Sala Llinas, Laura Sebastián, Javier Segovia Cubero, María Teresa Subirana Domenech, REHAP Consortium, Ana Argibay, Maria Baldà Masmiquel, Eduardo Callejas Moraga, Antonio-J. Chamorro, Dolores Colunga-Argüelles, Vicent Fonollosa-Pla, Mayka Freire, Cristina González-Echávarri, Alfredo Guillén-del-Castillo, Maria Teresa Herranz Marín, Ana Belén Madroñero Vuelta, Adela Marín Ballvé, Norberto Ortego-Centeno, Melany Pestaña Fernández, Xavier Pla Salas, Ignasi Rodríguez Pintó, Manuel Rubio-Rivas, Luis Sáez Comet, Gonzalo Salvador Cervelló, Carmen Pilar Simeón-Aznar, José Antonio Todolí Parra, Carles Tolosa-Vilella, Luis Trapiella, José Antonio Vargas Hitos

**Affiliations:** 1 Unit of Autoimmune Diseases, Department of Internal Medicine, Hospital Universitario Vall d’Hebron, Barcelona, Spain; 2 Pneumology Department, Hospital Universitario Vall d’Hebrón, Passeig Vall d’Hebron 119-129, 08035 Barcelona, Spain; 3 Physiology Department, Universitat Autònoma de Barcelona, Barcelona, Spain; 4 Department of Internal Medicine, Hospital Universitario Central de Asturias, Oviedo, Asturias Spain; 5 Unit of Autoimmune Diseases, Department of Internal Medicine, Hospital Universitario de Bellvitge-IDIBELL, L’Hospitalet de Llobregat, Barcelona, Spain; 6 Pulmonary Hypertension Unit, Cardiology Department of Hospital, Universitario, 12 de Octubre, Madrid, Spain; 7 Unit of Systemic Autoimmune Diseases and Thrombosis, Department of Internal Medicine, Complejo Hospitalario Universitario de Vigo, Vigo, Pontevedra Spain; 8 Pulmonary Medicine Department, Hospital Clínic de Barcelona/Institut d’Investigacions Biomèdiques August Pi i Sunyer (IDIBAPS), Barcelona, Spain; 9 Centro de Investigación Biomédica en Red de Enfermedades Respiratorias (CIBERES), Madrid, Spain; 10 Unit of Systemic Autoimmune Diseases, Department of Internal Medicine, Consorci Hospitalari de Vic, Vic, Barcelona Spain; 11 Pneumology Department, Hospital Universitario Marqués de Valdecilla, Santander, Cantabria Spain; 12 Department of Internal Medicine, Hospital General San Jorge, Huesca, Spain; 13 Cardiology Department, Hospital Universitario de Canarias, Santa Cruz de Tenerife, Spain; 14 Department of Internal Medicine, Hospital Universitario Miguel Servet, Zaragoza, Spain; 15 Pneumology Department, Hospital Universitario Miguel Servet, Zaragoza, Spain; 16 Autoimmune Diseases Research Unit, Department of Internal Medicine, Biocruces Bizkaia Health Research Institute, Hospital Universitario Cruces, University of the Basque Country, Barakaldo, Spain; 17 Cardiology Department, Hospital Universitario Gregorio Marañón, Madrid, Spain; 18 Inst Invest Biosanitaria Ibs Granada, Department of Internal Medicine, Unit of Systemic Autoimmune Diseases, Hospital Universitario San Cecilio, Granada, Spain; 19 Department of Medicine, Facultad de Medicina, Hospital Universitario San Cecilio, Granada, Spain; 20 Pneumology Department, Hospital Clínico Universitario de Valencia, Valencia, Spain; 21 Department of Internal Medicine, Parc Taulí, Hospital Universitario, Sabadell, Barcelona Spain; 22 Centro de Investigación Biomédica en Red de Enfermedades Cardiovasculares (CIBERCV)/Instituto de Salud Carlos III, Madrid, Spain; 23 Pneumology Department, Hospital Universitario Basurto, Bilbao, Spain; 24 Cardiology Department, Hospital Universitario Rey Juan Carlos, Móstoles, Madrid, Spain; 25 Pneumology Department, Hospital Universitario Central de Asturias, Oviedo, Spain; 26 Unitat Integrada de Cardiopaties Congènites de l’Adolescent i de l’Adult Vall d’Hebron-Sant Pau, Department of Cardiology, Vall d’Hebron University Hospital and CIBERCV, Barcelona, Spain; 27 Unidad Médico-Quirúrgica de Enfermedades Respiratorias, Instituto de Biomedicina de Sevilla (IBiS), Pneumology Department, Hospital Universitario Virgen del Rocío, Sevilla, Spain; 28 Internal Medicine Department, Hospital Universitario Virgen del Rocío, Sevilla, Spain; 29 Pneumology Department, Hospital General Universitario de Alicante, Alicante, Spain; 30 Department of Cardiology, Hospital Universitario Puerta de Hierro-Majadahonda, Madrid, Spain; 31 Department of Cardiology, Hospital Universitario Río Hortega, Valladolid, Spain; 32 Pneumology Department, Hospital Universitario Virgen del Rocío, Sevilla, Spain; 33 Internal Medicine Department, Hospital Jerez de la Frontera, Jerez de la Frontera, Cádiz, Spain; 34 Cardiology Department, Hospital Virgen de la Salud, Toledo, Spain; 35 Cardiology Department, Hospital Universitario Miguel Servet, Zaragoza, Spain; 36 Pneumology Department, Hospital Universitario y Politécnico La Fe, Valencia, Spain; 37 Pneumology Department, Hospital Universitario A Coruña, A Coruña, Spain; 38 Pneumology Department, Hospital Universitario Fundación Jiménez Díaz, Madrid, Spain; 39 Cardiology Department, Hospital Universitario La Fe, Valencia, Spain; 40 Centro de Investigación Biomédica en Red de Enfermedades Cardiovasculares (CIBERCV), Madrid, Spain; 41 Pneumology Department, Hospital Universitario Son Espases, Palma, Islas Baleares Spain; 42 Pulmonary Medicine Department, Hospital Josep Trueta, Gerona, Spain; 43 Department of Cardiology, Hospital Universitario Puerta de Hierro-Majalahonda, Madrid, Spain; 44 Unitat Integrada de Cardiopaties Congènites de l’Adolescent I de l’Adult Vall d’Hebron-Sant Pau, Department of Cardiology, Vall d’Hebron University Hospital, Barcelona, Spain; 45 Department of Internal Medicine, Hospital Clínico Universitario de Salamanca, Universidad de Salamanca-IBSAL, Salamanca, Spain; 46 Unit of Autoimmune Diseases, Department of Internal Medicine, Hospital Clínico Universitario de Santiago, Santiago de Compostela, A Coruña, Spain; 47 Department of Internal Medicine, Hospital General Universitario J.MMorales Meseguer, Murcia, Spain; 48 Unit of Autoimmune Diseases, Department of Internal Medicine, Hospital Clínico Universitario Lozano Blesa, IIS Aragón, Zaragoza, Spain; 49 Department of Internal Medicine, Hospital Universitario Mútua Terrassa, Terrassa, Barcelona Spain; 50 Department of Internal Medicine, Hospital de Manises, Manises, Valencia Spain; 51 Department of Internal Medicine, Hospital Universitario y Politécnico La Fe, Valencia, Spain; 52 Systemic Autoimmune Diseases Unit, Department of Internal Medicine, Hospital Universitario Virgen de las Nieves, Granada, Spain

Correction to: *Scientific Reports* 10.1038/s41598-022-09353-z, published online 28 March 2022

The original version of this Article contained an error in the spelling of the author Joan Albert Barberá which was incorrectly given as Joan Albert Barberá Mir.

The original Article has been corrected.

