# Supplementary material for: Impact of interstitial lung disease on the survival of systemic sclerosis with pulmonary arterial hypertension
Source: Sci Rep. 2022 Mar 28;12:5289. doi: 10.1038/s41598-022-09353-z (PMC8960788; doi:10.1038/s41598-022-09353-z)
Supplement: Supplementary file 1 — Supplementary Information. [file 41598_2022_9353_MOESM1_ESM.docx]

**SUPPLEMENTARY MATERIAL**

**Appendix A**

**Coordinators of the REHAP Registry:**  Dr. Pilar Escribano Subías (Spain)

Dr. Isabel Blanco (Spain)

Members of the REHAP Group are:

Aurtenetxe Pérez A, Barbera JA, Barrios Garrido-Lestache E, Bedate P, Blanco I, Cifrián JM, Cristo Ropero MJ, Domingo JA, Dos L, Elías Hernández T, Escribano-Subías P, García Hernández FJ, Gil Carbonell J, González Segovia A, Hermida Valverde T, Hernández Baldomero IF, Hernández-González I, Herrero Huertas J, Jara Palomares L, Jiménez Arjona J, Lara A, Lázaro-Salvador M, López-Meseguer M, López-Ramón M, López-Reyes R, Marín González M, Martínez Meñaca A, Mazo Etxaniz FJ, Mombiela T, Naranjo Velasco V, Otero Candelera R, Otero González I, Revilla-López E, Rodríguez Lozano B, Rodríguez Nieto MJ, Rueda J, Sáez Giménez B, Safont B, Sala E, Sebastián L, Segovia Cubero J, Subirana MT

**Appendix B**

**Coordinator of the RESCLE Registry:** Dr. Carmen Pilar Simeón Aznar (Spain)

Members of the RESCLE Group are:

Argibay A, Baldà M, Callejas Moraga E, Chamorro AJ, Colunga D, Fonollosa V, Freire M, González-Echávarri C, Guillén-Del-Castillo A, Herranz Marín MT, Madroñero AB, Marín Ballvé A, Ortego-Centeno N, Pestaña M, Pla Salas X, Rodríguez Pintó I, Rubio Rivas M, Sáez Comet L, Salvador Cervelló G, Simeón CP, Todolí Parra JA, Tolosa C, Trapiella L, Vargas Hitos JA

**Supplementary table I. Equivalences in causes of death**

| **REHAP registry**  **(PAH-SSc)** | **RESCLE registry**  **(Non-PAH-SSc)** | **RESCLE-REHAP** |
| --- | --- | --- |
| Heart failure  Sudden death | PAH-related (excluded) | **PAH-related** |
| Haemoptysis | PAH + ILD-related (excluded) |  |
| Underlying disease | SSc-related:  ILD  SRC  Digestive  Coronary heart disease  Chronic renal failure  Heart failure  Arrhythmia | **SSc-related** |
| Malignancy | Malignancy | **Malignancy** |
| - | Heart failure* | **Heart failure** |
| Infection | Sepsis | **Infection** |
| Pulmonary thromboembolism | Pulmonary thromboembolism | **Pulmonary thromboembolism** |
| Acute myocardial infarction | Coronary heart disease | **Coronary heart disease** |
| Stroke | Stroke | **Stroke** |
| Other  Unknown  Bleeding (No haemoptysis) | COPD  Other | **Other** |

COPD, chronic obstructive pulmonary disease; ILD, interstitial lung disease; PAH, pulmonary arterial hypertension; SRC, scleroderma renal crisis; SSc, systemic sclerosis.

*Other causes than PAH

**Supplementary table II.** **Demographic, clinical, and echocardiography data of patients with ILD. PAH-SSc patients (REHAP) are compared with non-PAH-SSc patients (RESCLE)**

|  | **Patients with ILD** | | | | |
| --- | --- | --- | --- | --- | --- |
|  | **N** | **PAH-SSc (REHAP)**  **N = 92** | **N** | **Non-PAH-SSc (RESCLE)**  **N=428** | **P-value** |
| Gender, female, n (%) | 92 | 75 (81.5) | 428 | 361 (84.3) | 0.532 |
| Age at diagnosis, years, mean (SD) | 92 | 62.1(11.8) | 428 | 50.9 (15.4) | **<0.001** |
| NYHA FC, n (%) | 92 |  | 399 |  |  |
| I-II | - | 22 (23.9) | - | 357 (89.5) | **<0.001** |
| III-IV | - | 70 (76.1) | - | 42 (10.5) | **<0.001** |
| Pulmonary function tests |  |  |  |  |  |
| FVC (%) predicted, mean (SD) | 88 | 70.9 (21.9) | 393 | 80.1 (20.8) | **<0.001** |
| <60%, n (%) | - | 35 (39.8) | - | 74 (17.3) | **<0.001** |
| 60% - <80%, n (%) | - | 20 (22.7) | - | 122 (31.0) | 0.155 |
| ≥80%, n (%) | - | 33 (37.5) | - | 197 (50.9) | **0.034** |
| DLCO (%) predicted, mean (SD) | 74 | 39.4 (17.0) | 317 | 65.2 (35.6) | **<0.001** |
| DLCO ≤55%, n (%) | - | 64 (86.5) | - | 111 (35.0) | **<0.001** |
| FVC/DLCO, mean (SD) | 74 | 2.2 (1.2) | 317 | 1.4 (0.5) | **<0.001** |
| FVC/DLCO ≥1.6, n (%) | - | 48 (64.9) | - | 88 (28.1) | **<0.001** |
| FVC/DLCO ≥1.4, n (%) | - | 54 (73.0) | - | 136 (43.4) | **<0.001** |
| Electrocardiogram |  |  |  |  |  |
| Arrhythmia/Atrial fibrillation, n (%) | 81 | 7 (8.6) | 203 | 14 (6.9) | 0.620 |
| Echocardiography |  |  |  |  |  |
| LVEF (%), mean (SD) | 64 | 63.9 (8.5) | 334 | 63.1 (6.9) | 0.509 |
| sPAP, mmHg, mean (SD) | 82 | 65.5 (20.4) | 184 | 30.5 (6.3) | **<0.001** |
| sPAP >40, n (%) | - | 77 (93.9) | - | 13 (7.1) | **<0.001** |
| Tricuspid regurgitation, yes, n (%) | 83 | 76 (91.6) | 318 | 145 (45.6) | **<0.001** |
| Mild | - | 35 (42.2) | - | 139 (43.7) | 0.804 |
| Moderate | - | 30 (34.9) | - | 5 (1.6) | **<0.001** |
| Severe | - | 11 (13.2) | - | - | **<0.001** |
| No | - | 7 (8.4) | - | 173 (54.4) | **<0.001** |
| TAPSE, mm, mean (SD) | 44 | 16.5 (5.3) | 58 | 20.5 (5.2) | **<0.001** |
| Pericardial effusion, n (%) | 83 | 20 (24.1) | 316 | 22 (7.0) | **<0.001** |

DLCO, diffusing capacity for carbon monoxide; FVC, forced vital capacity; ILD, interstitial lung disease; LVEF, left ventricular ejection fraction; NYHA FC, New York Heart Association functional class; sPAP, systolic pulmonary artery pressure; SD, standard deviation; TAPSE, tricuspid annular plane systolic excursion.

**Supplementary table III.** **Demographic, clinical, and echocardiography data of patients without ILD. PAH-SSc patients (REHAP) are compared with non-PAH-SSc patients (RESCLE)**

|  | **Patients without ILD** | | | | |
| --- | --- | --- | --- | --- | --- |
|  | **N** | **PAH-SSc (REHAP)**  **N = 128** | **N** | **Non-PAH - SSc (RESCLE)**  **N = 517** | **P-value** |
| Gender, female, n (%) | 128 | 117 (91.4) | 511 | 453 (88.6) | 0.428 |
| Age at diagnosis, years, mean (SD) | 128 | 63.8 (11.4) | 509 | 50.9 (15.2) | **<0.001** |
| NYHA FC, n (%) | 128 |  | 155 |  |  |
| I-II | - | 4 (3.1) | - | 147 (94.8) | **<0.001** |
| III-IV | - | 42 (32.8) | - | 8 (5.2) | **<0.001** |
| Pulmonary function tests |  |  |  |  |  |
| FVC (%) predicted, mean (SD) | 113 | 86.2 (18.6) | 412 | 97.1 (15.9) | **<0.001** |
| <60%, n (%) | - | 6 (5.3) | - | 2 (0.5) | **0.002** |
| 60% - <80%, n (%) | - | 39 (34.5) | - | 46 (11.0) | **<0.001** |
| ≥80%, n (%) | - | 68 (60.2) | - | 364 (87.1) | **<0.001** |
| DLCO (%) predicted, mean (SD) | 99 | 49.1 (17.9) | 309 | 86.3 (46.6) | **<0.001** |
| DLCO ≤55%, n (%) | - | 71 (71.7) | - | 23 (7.3) | **<0.001** |
| FVC/DLCO, mean (SD) | 93 | 2.0 (0.8) | 304 | 1.3 (0.4) | **<0.001** |
| FVC/DLCO ≥1.6, n (%) | - | 63 (67.7) | - | 50 (16.2) | **<0.001** |
| FVC/DLCO ≥1.4, n (%) | - | 73 (78.5) | - | 95 (30.8) | **<0.001** |
| Electrocardiogram |  |  |  |  |  |
| Arrhythmia/Atrial fibrillation, n (%) | 119 | 11 (9.2) | 249 | 10 (3.9) | 0.054 |
| Echocardiography |  |  |  |  |  |
| LVEF (%), mean (SD) | 95 | 64.3 (8.2) | 353 | 64.4 (6.5) | 0.855 |
| sPAP, mmHg, mean (SD) | 116 | 69.6 (22.5) | 224 | 29.5 (5.9) | **<0.001** |
| sPAP >40, n (%) | - | 114 (98.3) | - | 14 (6.3) | **<0.001** |
| Tricuspid regurgitation, yes, n (%) | 106 | 102 (96.2) | 346 | 181 (52.0) | **<0.001** |
| Mild | - | 40 (37.7) | - | 177 (50.6) | **0.019** |
| Moderate | - | 47 (44.3) | - | 4 (1.2) | **<0.001** |
| Severe | - | 15 (14.1) | - | - | - |
| No | - | 4 (3.8) | - | 165 (48.0) | **<0.001** |
| TAPSE, mm, mean (SD) | 60 | 18.9 (4.8) | 52 | 18.9 (8.4) | 0.964 |
| Pericardial effusion, n (%) | 104 | 30 (28.8) | 342 | 12 (3.5) | **<0.001** |

DLCO, diffusing capacity for carbon monoxide; FVC, forced vital capacity; ILD, interstitial lung disease; LVEF, left ventricular ejection fraction; NYHA FC, New York Heart Association functional class; sPAP, systolic pulmonary artery pressure; SD, standard deviation; TAPSE, tricuspid annular plane systolic excursion.

**Supplementary table IV. Causes of death in PAH-SSc and non-PAH-SSc patients**

| **Causes of death, N (%)** | **PAH-SSc**  **(n=186)** | **non-PAH-SSc (n=185)** | **P-value** |
| --- | --- | --- | --- |
| PAH-related | 112 (60.0) | - | **<0.001** |
| SSc-related | 16 (8.6) | 45 (24.3) | **<0.001** |
| Malignancy | 7 (3.8) | 33 (17.8) | **<0.001** |
| Heart failure | - | 15 (8.1) | - |
| Infection | 14 (7.5) | 14 (7.6) | 0.846 |
| Pulmonary thromboembolism | 1 (0.5) | 3 (1.6) | 0.354 |
| Coronary heart disease | - | 5 (2.7) | - |
| Stroke | 1 (0.5) | 3 (1.6) | 0.354 |
| Other | 35 (18.8) | 54 (29.2) | **0.007** |
| Unknown | - | 13 (7.0) | **-** |

COPD, obstructive chronic pulmonary disease; PAH, pulmonary arterial hypertension; ILD, interstitial lung disease; SSc, systemic sclerosis

**Supplementary table V.** **Demographic, clinical, and hemodynamic data of patients with PAH-SSc and concomitant ILD according to FVC impairment**

|  | **N** | **PAH-SSc**  **with ILD and**  **FVC <60% (n=35; 40%)** | **N** | **PAH-SSc**  **with ILD and FVC ≥60%**  **(n=53; 60%)** | **P-value** |
| --- | --- | --- | --- | --- | --- |
| Gender, female, n (%) | 35 | 28 (80.0) | 53 | 43 (81.0) | 1.000 |
| Age at PAH diagnosis, years, mean (SD) | 35 | 57.8 (13.8) | 53 | 64.7 (10.0) | **0.009** |
| NYHA FC, n (%) | 35 |  | 53 |  |  |
| I-II | - | 8 (22.8) | - | 14 (26.4) | 0.804 |
| III-IV | - | 27 (77.1) | - | 39 (73.6) | 0.804 |
| 6MWT, meters, mean (SD) | 30 | 306.1 (130.4) | 46 | 272.5 (148.6) | 0.315 |
| Hemodynamics, mean (SD)  RAP, mm Hg  SvO2, %  CO, L/min  CI, L/min/m^2^  PVR, Wood units  mPAP, mm Hg | 35  25  35  31  35  35 | 8.9 (5.2)  66.0 (9.5)  4.3 (1.3)  2.5 (0.6)  8.0 (4.8)  38.3 (12.8) | 52  27  52  49  52  53 | 7.2 (4.3)  65.1 (6.9)  3.9 (1.2)  2.3 (0.6)  8.4 (5.3)  38.0 (10.2) | 0.098  0.699  0.144  0.091  0.690  0.897 |
| Pulmonary function tests |  |  |  |  |  |
| DLCO (%) predicted, mean (SD) | 26 | 40.0 (18.8) | 48 | 39.0 (17.8) | 0.811 |
| DLCO ≤55%, n (%) | - | 22 (85.0) | - | 42 (88.0) | 0.734 |
| FVC/DLCO, mean (SD) | 26 | 1.5 (0.7) | 48 | 2.6 (1.3) | **<0.001** |
| FVC/DLCO ≥1.6, n (%) | - | 9 (35.0) | - | 39 (81.0) | **<0.001** |
| FVC/DLCO ≥1.4, n (%) | - | 12 (46.0) | - | 42 (88.0) | **<0.001** |
| Biomarkers, median (IQR)  NTproBNP, pg/mL    BNP, pg/mL | 35  35 | 340.0  (239.0-5,575.0)  255.0  (80.0-524.0) | 53  53 | 1,370.0  (347.0-3,703.5)  232.0  (48.0-967.0) | 0.809  0.332 |
| Electrocardiogram |  |  |  |  |  |
| Arrhythmia/Atrial fibrillation, n (%) | 31 | 4 (13.0) | 47 | 2 (4.3) | 0.208 |
| Echocardiography |  |  |  |  |  |
| LVEF (%), mean (SD) | 26 | 64.0 (9.1) | 34 | 63.9 (7.9) | 0.947 |
| sPAP, mmHg, mean (SD) | 31 | 66.1 (20.0) | 47 | 65.5 (21.2) | 0.907 |
| sPAP >40, n (%) | - | 29 (93.5) | - | 44 (93.6) | 1.000 |
| Tricuspid regurgitation, yes, n (%) | 33 | 29 (87.9) | 47 | 44 (93.6) | 0.439 |
| Mild | - | 11 (33.3) | - | 22 (46.8) | 0.256 |
| Moderate | - | 15 (45.4) | - | 15 (31.9) | 0.247 |
| Severe | - | 3 (9.1) | - | 7 (14.9) | 0.512 |
| No | - | 4 (12.1) | - | 3 (6.4) | 0.439 |
| TAPSE, mm, mean (SD) | 12 | 15.8 (4.2) | 30 | 17.0 (5.8) | 0.519 |
| Pericardial effusion, n (%) | 32 | 6 (19.0) | 47 | 13 (28.0) | 0.429 |
| PAH-targeted treatments at diagnosis | 35 |  | 53 |  |  |
| No treatment  Monotherapy | - | 0 (0.0)  17 (48.6) | - | 4 (7.5)  14 (26.4) | 0.148  **0.042** |
| Up-front combination | - | 18 (51.4) | - | 35 (66.0) | 0.189 |

BNP, B-type natriuretic peptide; CI, Cardiac index; CO, Cardiac output; DLCO, diffusing capacity for carbon monoxide; FVC, forced vital capacity; IQR: interquartile range; ILD, interstitial lung disease; LVEF, left ventricular ejection fraction; mPAP, mean pulmonary artery pressure; NTproBNP, N-terminal pro B-type natriuretic peptide; NYHA FC, New York Heart Association functional class; PVR, pulmonary vascular resistance; RAP, right atrial pressure; sPAP, systolic pulmonary artery pressure; SD, standard deviation; SvO_2_, mixed venous oxygen saturation; TAPSE, tricuspid annular plane systolic excursion; 6MWT, 6-minute walking test.
